# How to Peer Review a Neurology Education Manuscript

**DOI:** 10.1212/NE9.0000000000200099

**Published:** 2023-11-06

**Authors:** Daniel G. Di Luca, Steven M. Lazar, Preeta Gupta, Marie Charmaine S. Lukban, Cole Crowson, Dara V.F. Albert, Jeremy J. Moeller, Andres Fernandez, Andrew M. Southerland, Zachary London

**Affiliations:** From the Department of Neurology (D.G.D.L.), Washington University School of Medicine, St. Louis, MO; Section of Pediatric Neurology and Developmental Neuroscience (S.M.L.), Department of Pediatrics, Texas Children's Hospital/Baylor College of Medicine, Houston; Department of Neurology (P.G., Z.L.), University of Michigan, Ann Arbor; Philippine General Hospital (M.C.S.L.), Manilla; Department of Neurology (C.C.), Oregon Health & Science University, Portland; Division of Neurology (D.V.F.A.), Department of Pediatrics, Nationwide Children's Hospital/The Ohio State University, Columbus; and Department of Neurology (J.J.M., A.F., A.M.S.), Yale School of Medicine, New Haven, CT.

## Abstract

Peer review is an essential process in scientific research, ensuring the comprehensiveness, accuracy, and suitability of manuscripts for publication. Neurology education research differs from biomedical clinical research in several ways. These differences encompass specific paradigms, the use of theoretical frameworks, and different methodological approaches. Despite the high number of studies and journal publications on neurology education, there is a dearth of resources and guidance on how to perform a formal review on this specific literature. This article aims to review the distinctive features of neurology education from clinical research while proposing an organizational framework and model for performing peer reviews of papers focused on neurology education.

## Introduction

Neurology education has emerged as a recognized discipline within the field of neurology.^1,2^ This has resulted in efforts to establish academic platforms for the dissemination of neurology education scholarship such as the journal, *Neurology*® *Education*. As the visibility of education scholarship in neurology continues to rise, there is a growing need for peer reviewers who possess the skills to understand, critically analyze, and provide practical recommendations to enhance the peer review publication process.^3-5^

A robust review process for neurology education scholarship is of enormous importance to the future of our field. The review process is intended to identify high-quality educational scholarship, improve the quality of scholarly dissemination, and identify situations of ethical conflicts in scholarly reporting. Therefore, a meticulous review process is essential to ensuring that this scholarship adheres to the highest level of quality and rigor.^6^

In this article, we present a practical introduction to key components of the peer review process for medical education (MedEd) scholarship. Our aim is to serve as a guiding reference for peer reviewers as they assess education-focused manuscripts, highlighting key distinctions compared with traditional biomedical research manuscripts. However, we acknowledge that these 2 forms of research can have common elements and shared principles in the peer review process.

### What Are the Different Characteristics of Journals Publishing MedEd Scholarship?

Academic journals that publish MedEd scholarship cater to diverse audiences. When reviewing a MedEd manuscript, it is essential to determine whether the report falls within the journal's scope and understand the perspectives of the target audience. For example, some journals, such as *Medical Education*, *Academic Medicine*, and *Medical Teacher*, target the general MedEd audience. These journals emphasize findings, insights, and theoretical understandings that have broader transferability and applicability.

General medical journals such as the *Journal of the American Medical Association*, the *New England Journal of Medicine*, and the *British Medical Journal* publish educational research alongside clinical research. Moreover, clinically oriented specialty journals such as *Neurology*, *Pediatrics*, and *Emergency Medicine* publish domain-specific educational research, often within a dedicated trainee subsection. Finally, journals such as *Neurology: Education* focus entirely on specialty-specific MedEd scholarship.

### Differences Between MedEd Research and Biomedical Research

Clinical and translational research traditionally accompanies a post-positivist paradigm, where systemic experimental inquiry aims to define a single reality or truth. Research within this paradigm is typically quantitative and characterized by statistical analysis and hypothesis testing. Educational and social science researchers often approach research through an interpretivist or constructivist lens in which reality and truth are dependent on the internal experiences and perspectives of individuals; it may be culturally derived, historically situated, and contextually dependent. This approach integrates the research environment and considers the participants' perspectives.^7,8^

One other key distinguishing feature between MedEd and Biomedical research is the clear use of theoretical framework in MedEd research. Theory provides an abstract description of the relationships between concepts, facilitating our understanding of the world. A theoretical framework, derived from one or more theories, is a “logically developed and interconnected set of concepts and premises” that researchers establish to support a study.^9^ In other words, theory is applied to research through a theoretical framework. A sample framework for evaluating the use of theory in MedEd scholarship has been adapted from McGrath et al. (Table 1).^10^ Incorporating a theoretical basis to MedEd research allows educators to place their findings in the context of existing literature and understand how, why, and in what way an educational intervention resulted in the observed outcomes.

**Table 1:**
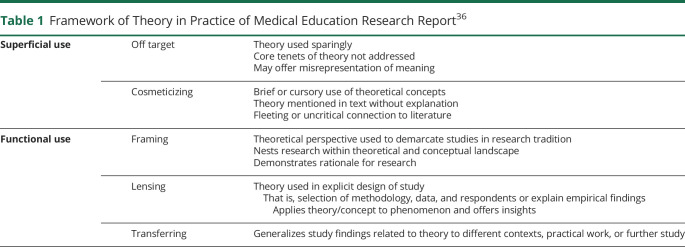
Framework of Theory in Practice of Medical Education Research Report^36^

Superficial use	Off target	Theory used sparingly Core tenets of theory not addressed May offer misrepresentation of meaning
	Cosmeticizing	Brief or cursory use of theoretical concepts Theory mentioned in text without explanation Fleeting or uncritical connection to literature
Functional use	Framing	Theoretical perspective used to demarcate studies in research tradition Nests research within theoretical and conceptual landscape Demonstrates rationale for research
	Lensing	Theory used in explicit design of study That is, selection of methodology, data, and respondents or explain empirical findings Applies theory/concept to phenomenon and offers insights
	Transferring	Generalizes study findings related to theory to different contexts, practical work, or further study

Both the post-positivist and interpretivist/constructivist paradigms come with their respective paradigmatic orientations that influence methodology and study designs. Classically, research in the post-positivist tradition is quantitative, leveraging experimental design, statistical comparisons, controls, and limiting of bias. Common methods of a post-positivist approach in MedEd include pre-post intervention assessment of knowledge and attitudes. Qualitative research within the interpretivist/constructivist tradition aims to understand beliefs, experiences, attitudes, behavior, and interactions of individuals. Common methods include interviews, focus groups, field notes, and observations. Mixed-methods research combines these traditions, using qualitative and quantitative methods to analyze data and provide unique insights. The choice of methodology in education research depends on the study's specific aims and is rooted in a relevant theoretical approach and paradigmatic orientation.

Other studies in MedEd encompass design and implementation, such as Design-based Research and Program Evaluation, which adopt a pragmatic approach to curricular and instructional design using both theoretical insights and methods that best serve the research question.^11^ Compared with other education subdisciplines, validated MedEd approaches specific to neurology are still undefined, and MedEd research in general remains a fluid and evolving field when compared with biomedical research (Table 2).

**Table 2:**
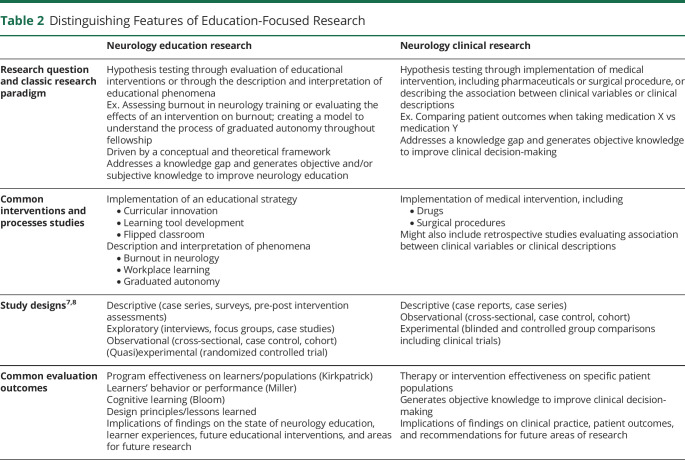
Distinguishing Features of Education-Focused Research

	Neurology education research	Neurology clinical research
Research question and classic research paradigm	Hypothesis testing through evaluation of educational interventions or through the description and interpretation of educational phenomena Ex. Assessing burnout in neurology training or evaluating the effects of an intervention on burnout; creating a model to understand the process of graduated autonomy throughout fellowship Driven by a conceptual and theoretical framework Addresses a knowledge gap and generates objective and/or subjective knowledge to improve neurology education	Hypothesis testing through implementation of medical intervention, including pharmaceuticals or surgical procedure, or describing the association between clinical variables or clinical descriptions Ex. Comparing patient outcomes when taking medication X vs medication Y Addresses a knowledge gap and generates objective knowledge to improve clinical decision-making
Common interventions and processes studies	Implementation of an educational strategy • Curricular innovation • Learning tool development • Flipped classroom Description and interpretation of phenomena • Burnout in neurology • Workplace learning • Graduated autonomy	Implementation of medical intervention, including • Drugs • Surgical procedures Might also include retrospective studies evaluating association between clinical variables or clinical descriptions
Study designs^7,8^	Descriptive (case series, surveys, pre-post intervention assessments) Exploratory (interviews, focus groups, case studies) Observational (cross-sectional, case control, cohort) (Quasi)experimental (randomized controlled trial)	Descriptive (case reports, case series) Observational (cross-sectional, case control, cohort) Experimental (blinded and controlled group comparisons including clinical trials)
Common evaluation outcomes	Program effectiveness on learners/populations (Kirkpatrick) Learners' behavior or performance (Miller) Cognitive learning (Bloom) Design principles/lessons learned Implications of findings on the state of neurology education, learner experiences, future educational interventions, and areas for future research	Therapy or intervention effectiveness on specific patient populations Generates objective knowledge to improve clinical decision-making Implications of findings on clinical practice, patient outcomes, and recommendations for future areas of research

### What Makes Peer Review of Methods in MedEd Unique?

While many publication houses such as Wolters Kluwer, Sage Publishing, Springer, and Web of Science (formerly Publons) provide training for reviewers, there is a need for resources tailored to reviewing MedEd scholarship.^10,12-16^ Peer review for MedEd, similar to any specialized field, requires familiarity and expertise in both content and common methodologic approaches. However, with exposure to the correct didactic materials, and under proper supervision, trainees have the potential to conduct independent peer reviews.

When approaching scholarship, reviewers must pay attention to the research paradigm to contextualize and evaluate the findings. Reviewers who have only been exposed to research through a post-positivist lens need to acquaint themselves with a broader range of research paradigms (e.g., constructivism) and methodologies (e.g., qualitative) to effectively assess MedEd publications.^17^ Given that, high‐quality MedEd research is grounded in theory within a theoretical framework, these should ideally be stated in the manuscript. Although variable, the description or discussion of the conceptual/theoretical basis of the study can usually be found in aims or methods.

Reviewers of MedEd scholarship need to be cognizant of concepts such as paradigms, educational theory, theoretical frameworks, conceptual frameworks, and methodologic frameworks.^9^ While these concepts also exist in traditional biomedical research, MedEd scholarship requires them to be clear and explicitly described to ensure a shared understanding of how the authors reached their conclusions. Furthermore, reviewing the application and use of the theory might provide additional insight of how it was incorporated into the MedEd manuscript and support the trustworthiness of the underlying assumptions and data described by the authors.

### Accepting or Declining an Invitation to Review

To avoid delays and long processing times in reaching a final decision and recommendation, it is important to promptly accept or decline an invitation to peer review. This expedites the review process and allows the journal to find a replacement reviewer, if needed. The reviewer should also consider whether the subject of the manuscript falls within their scope of expertise, particularly important for MedEd research, and determine whether they can complete the assigned review within the journal's deadline. Similar to all forms of peer review, it is important for the reviewer to reflect on any potential conflict of interests and ensure that they can provide an unbiased assessment of the manuscript. Specifically, the reviewer should decline and disclose any potential conflicts. When uncertain, it is recommended to directly communicate with the editor or journal staff to discuss any potential conflicts.

## Content, Results, and Discussion

The organization of a peer review for a manuscript focused on MedEd follows a similar structure to biomedical research articles (Table 3). The reviewer's main task is to critically evaluate the paper and provide constructive comments to enhance the manuscript. During this process, the reviewer should also identify any potential flaws and discuss broader issues such as validity and generalizability (in quantitative research) or trustworthiness and transferability (in qualitative research).^18^ Similarly, the appropriateness of the statistical analysis and other methodological considerations should be evaluated. The results should also be reviewed in detail to ensure that the findings are clearly stated in a logical sequence and in line with the conclusions.

**Table 3:**
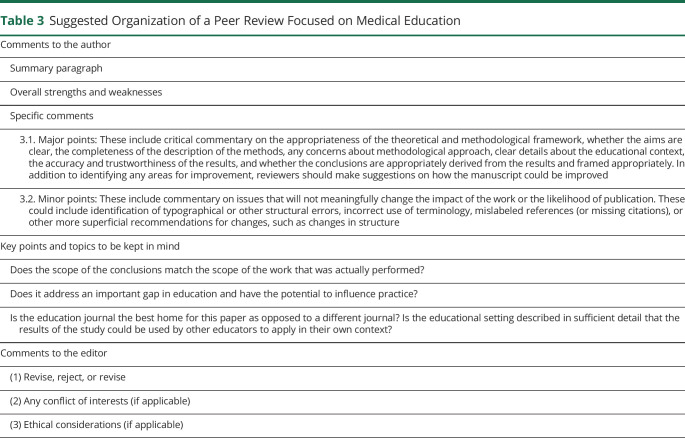
Suggested Organization of a Peer Review Focused on Medical Education

Comments to the author
Summary paragraph
Overall strengths and weaknesses
Specific comments
3.1. Major points: These include critical commentary on the appropriateness of the theoretical and methodological framework, whether the aims are clear, the completeness of the description of the methods, any concerns about methodological approach, clear details about the educational context, the accuracy and trustworthiness of the results, and whether the conclusions are appropriately derived from the results and framed appropriately. In addition to identifying any areas for improvement, reviewers should make suggestions on how the manuscript could be improved
3.2. Minor points: These include commentary on issues that will not meaningfully change the impact of the work or the likelihood of publication. These could include identification of typographical or other structural errors, incorrect use of terminology, mislabeled references (or missing citations), or other more superficial recommendations for changes, such as changes in structure
Key points and topics to be kept in mind
Does the scope of the conclusions match the scope of the work that was actually performed?
Does it address an important gap in education and have the potential to influence practice?
Is the education journal the best home for this paper as opposed to a different journal? Is the educational setting described in sufficient detail that the results of the study could be used by other educators to apply in their own context?
Comments to the editor
(1) Revise, reject, or revise
(2) Any conflict of interests (if applicable)
(3) Ethical considerations (if applicable)

In contrast to the peer review process in the biomedical research model, reviews in MedEd should take into account practical considerations related to the situated/contextual elements of the interpretivist/constructivist approach. Because the results are highly influenced by the specific educational setting, learners, and topic being studied, it is crucial for reviewers to thoroughly analyze these details to determine the applicability of the insights to other settings. In addition, reviewers should ensure that that the authors have aligned their methods, results, and conclusions with their chosen research paradigm and methodology. For instance, if authors adopt a constructivist/interpretivist paradigm, their conclusions should not make positivist statements (e.g., “Our results show that this curriculum leads to improvement in medical student knowledge” or “given these findings, this curriculum is readily generalizable”). Alternatively, their conclusions should be in line with the constructivist/interpretivist paradigm (e.g., “Our results show that third-year medical students at our institution demonstrated growth in their knowledge, and important contributors to this knowledge growth included…” or “our findings support the transferability of XYZ in context A due to …”).

### Comments to the Author

The journal guidelines should be thoroughly reviewed before completing the review. In general, editors are looking for a comprehensive understanding of the paper.^19-21^ The overall message of the paper holds greater importance than minor details that do not change the main take-away points or require mere grammatical corrections. The reviewer should reflect on whether the content and focus of the paper is suitable for the journal's subject area, the intended audience, and the stated objectives or aims of the paper itself.^10^ It is important to keep the comments and suggestions well-organized and easy to follow. A common recommendation is to begin with a brief summary of the article, followed by addressing limitations and providing specific recommendations to enhance the quality of the submission.

Some reviewers choose to categorize their comments as either major or minor recommendations. Major recommendations address key issues that inform the overall message of the article. Examples include flaws that necessitate a major revision in methodology or require additional details, especially additional information about the educational context (a commonly missing item in MedEd work as previously outlined). Other major recommendations may include the need to acknowledge additional limitations or explain a disconnect between the findings and conclusions. Minor recommendations pertain to noncritical minor issues, such as grammar, organization of the structure of the manuscript, missing or mislabeled citations, or misuse of an educational term (e.g., “competence” vs “competency”).^15,22^

In addition, reviewers should evaluate whether the manuscript addresses an important gap in education and whether it has the potential to change practice or improve what is known about the topic. In quantitative research, external validity and issues related to reproducibility and generalizability hold importance. For instance, if the manuscript addresses the influence of an educational resource that is not widely available or scalable, readers may not be able to benefit from the intervention, or it would be unlikely to be incorporated in a curriculum. For qualitative research, the authors' reflexivity, including their prior experiences, perspectives, and assumptions, can influence the research process.^23^

### Confidential Comments to the Editor

Here, reviewers should explicitly state their final recommendation for the paper, indicating whether it should be published, rejected, or revised (minor or major). In this section, the reviewers should also discuss any ethical issues or concerns related to the paper, including plagiarism or potential violations of ethical conduct in research. A concise, high-level summary of the manuscript might also be helpful to be included. Finally, the reviewer should disclose any conflict of interest that might have biased their assessment of the paper or whether the manuscript was coreviewed with a colleague.^15,22^

### Peer Review Bias

Reviewers must be aware of potential sources of bias and take steps to mitigate them. Explicit and implicit bias can occur in the research itself, and the biases of the reviewer can affect whether the work is published, thus amplifying the effect of the bias. Explicit bias arises from conscious thought and can be deliberately regulated. Explicit bias hinders impartial review and constitutes a conflict of interest.

On the other hand, an unconscious bias refers to attitudes, stereotypes, motivations, or assumptions that can occur without one's knowledge, control, or intention. Examples of unconscious bias include gender bias, cultural bias, age bias, language bias, and institutional bias.^19^ Other common types of bias include selection, retrieval, and expectancy bias among others.^24-26^ Recognizing and addressing these biases are crucial when quality, relevance, and competence are being evaluated.^27,28^

### Other Suggested Resources for Additional Learning

Various resources are available for reviewers of all experience levels, offering opportunities to enhance their understanding of the peer review process and improve their skills. One notable resource is the Web of Science online peer review course (former Publons Academy), which provides instruction on the fundamentals of peer review and allows reviewers to track and receive credit for their completed reviews.^29^ If permitted by the journal's editorial leadership, pairing mentor and mentee reviewers can be an effective strategy for developing expertise in MedEd review. If cases where a review is assisted, it is important to disclose this information to the editor. The American Academy of Neurology and the international Movement Disorder Society have developed mentored peer review programs specifically tailored to trainees, providing them with the opportunity to obtain individualized feedback and support from more experienced reviewers or editors.^6,30^

Publication guidelines have been developed for the many methods of scientific inquiry, including MedEd. Noteworthy resources include guidelines such as Standards for QUality Improvement Reporting Excellence in Education, which offer structured guidance for conducting education research.^31^ Additional models such as Guideline for Reporting Evidence-based practice Educational interventions and Teaching and Defined Criteria To Report INnovations in Education are listed in Table 4.^32,33^ Peer reviewers should be familiar with these guidelines to help regulate the publications in MedEd to align with evidence-based and expert-determined standards. In addition, there are existing scales available for assessing the quality of peer reviews. While these scales are not standardized or supported by qualitative or quantitative data, some journals might choose to adopt them.^34,35^

**Table 4:**
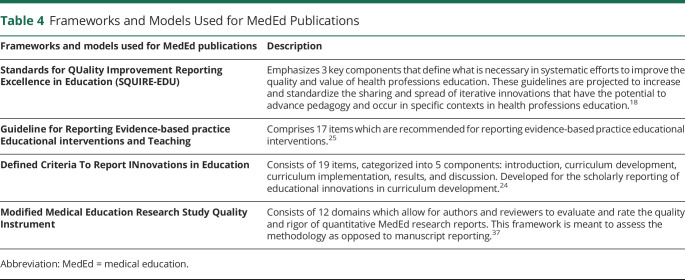
Frameworks and Models Used for MedEd Publications

Frameworks and models used for MedEd publications	Description
Standards for QUality Improvement Reporting Excellence in Education (SQUIRE-EDU)	Emphasizes 3 key components that define what is necessary in systematic efforts to improve the quality and value of health professions education. These guidelines are projected to increase and standardize the sharing and spread of iterative innovations that have the potential to advance pedagogy and occur in specific contexts in health professions education.^18^
Guideline for Reporting Evidence-based practice Educational interventions and Teaching	Comprises 17 items which are recommended for reporting evidence-based practice educational interventions.^25^
Defined Criteria To Report INnovations in Education	Consists of 19 items, categorized into 5 components: introduction, curriculum development, curriculum implementation, results, and discussion. Developed for the scholarly reporting of educational innovations in curriculum development.^24^
Modified Medical Education Research Study Quality Instrument	Consists of 12 domains which allow for authors and reviewers to evaluate and rate the quality and rigor of quantitative MedEd research reports. This framework is meant to assess the methodology as opposed to manuscript reporting.^37^

Abbreviation: MedEd = medical education.

## Conclusion

We hope that this article will empower both novice and experienced neurology educators to confidently engage in peer review of neurology education research. Recognizing that education research may differ from traditional clinical or biomedical research paradigms, peer reviewers need a unique academic lens to review neurology education manuscripts.

The field of neurology education research is experiencing exponential growth, necessitating the involvement of seasoned reviewers to uphold the highest standards of published scholarship and maximize its impact on our field. Including neurology trainees in the peer review process offers an accessible pathway to expand the pool of reviewers and mentor the next generation of medical educators, while formal mentored review can further cultivate educators' proficiency in the art of reviewing education research. Neurology education scholarship is a cornerstone of the future of neurology education, and innovations in neurology education are essential to ongoing improvements in patient care. By providing our peer reviewers with the knowledge and skills to vet and optimize educational scholarly work, we actively contribute to the continuous improvement of neurology as a whole.

## Appendix Authors

**Table:**

Name	Location	Contribution
Daniel G. Di Luca, MD, MSc	Department of Neurology, Washington University School of Medicine, St. Louis, MO	Drafting/revision of the manuscript for content, including medical writing for content; study concept or design
Steven M. Lazar, MD, MEd	Section of Pediatric Neurology and Developmental Neuroscience, Department of Pediatrics, Texas Children's Hospital/Baylor College of Medicine, Houston	Drafting/revision of the manuscript for content, including medical writing for content; study concept or design
Preeta Gupta, MD	Department of Neurology, University of Michigan, Ann Arbor	Drafting/revision of the manuscript for content, including medical writing for content; study concept or design
Marie Charmaine S. Lukban, MD, MBA	Philippine General Hospital, Manilla	Drafting/revision of the manuscript for content, including medical writing for content
Cole Crowson, MD	Department of Neurology, Oregon Health & Science University, Portland	Drafting/revision of the manuscript for content, including medical writing for content; study concept or design
Dara V.F. Albert, DO, MEd	Division of Neurology, Department of Pediatrics, Nationwide Children's Hospital/The Ohio State University, Columbus	Drafting/revision of the manuscript for content, including medical writing for content; study concept or design
Jeremy J. Moeller, MD, MSc	Department of Neurology, Yale School of Medicine, New Haven, CT	Drafting/revision of the manuscript for content, including medical writing for content; study concept or design
Andres Fernandez, MD, MSEd, FRCP(C)	Department of Neurology, Yale School of Medicine, New Haven, CT	Drafting/revision of the manuscript for content, including medical writing for content; study concept or design
Andrew M. Southerland, MD, MSc	Department of Neurology, Yale School of Medicine, New Haven, CT	Drafting/revision of the manuscript for content, including medical writing for content; study concept or design
Zachary London, MD	Department of Neurology, University of Michigan, Ann Arbor	Drafting/revision of the manuscript for content, including medical writing for content; study concept or design

## Glossary

MedEd: medical education
